# Verrucae pedis in children with juvenile idiopathic arthritis and other paediatric rheumatic diseases: a cross-sectional study

**DOI:** 10.1186/s13047-022-00526-7

**Published:** 2022-04-21

**Authors:** Jill Ferrari

**Affiliations:** University of East London, Stratford, London, UK

**Keywords:** Verruca Pedis, Juvenile idiopathic arthritis, Children, Plantar warts

## Abstract

**Background:**

Verrucae pedis (verrucae / VPs) are a common viral infection of the skin seen in children. There are limited studies of the prevalence, duration and impact of verrucae pedis in children who are immunosuppressed. The studies available suggest that, in these children, the warts are more widespread and are more long-standing. The primary aim of this study was to determine the prevalence of verrucae pedis in children attending rheumatology clinics who may have some degree of immunosuppression due to their prescribed medication and compare this to the reported prevalence in the healthy population.

**Method:**

Children attending out-patient rheumatology appointments were recruited. The young people were aged between four and 17 years old. A visual inspection of both feet was used to identify potential verrucae. Diagnosis of a verruca pedis was confirmed on observation of the typical clinical features. The location, duration of presence, previous treatments, presence of verrucae in other family members and psychological impact was recorded.

**Results:**

A total of 71 children were included. Of the group, 55 children had no verrucae present, 16 children had one or more verrucae. The prevalence of verrucae was 22.5%. Medication impacting on the immune system was prescribed in 80% of the group. There appeared to be no greater chance of having verrucae if taking immunosuppressive medication than compared to having no medication (OR = 1.1, 95%CI 0.26 to 4.48, *p* = 0.46). Children with verrucae tended to be between 9 and 12 years old. In total, 37.5% of the young people with verrucae had lesions reportedly present for 24 months or more. Two-thirds of the participants were not concerned about verrucae being present and most participants with a verruca were not aware of what a verruca was, but despite this the majority of participants (81%) had sought treatment for the verrucae.

**Conclusion:**

Children with Juvenile Idiopathic Arthritis and other rheumatic conditions have no greater prevalence of verrucae compared to the general population. The verrucae present were of a similar clinical type and did not seem to be more widespread or have atypical features, which has been reported in other immunocompromised populations. The percentage of lesions remaining beyond 24 months was found to be slightly greater than has been reported in other healthy populations. The children in this study seemed to be less emotionally concerned about their verrucae, despite this most families had sought treatment for the verrucae.

## Background

Verrucae pedis (verruca / verrucae / plantar warts) are a common viral infection of the skin that leads to the formation of benign, hyperkeratotic papillomas. Caused by the human papilloma virus (HPV), they are most prevalent in children, with a Dutch study suggesting that foot and hand warts had a prevalence of 33% in children aged 4–12 years, with 70% of these being plantar warts [1]. The study identified that warts tended towards natural resolution, with half the group being clear of warts after one year, agreeing with the findings of a previous study [2]. Younger children and those with non-Caucasian skin showed the highest resolution rates. In contrast, in young adults, the prevalence is much lower with a large Chinese study identifying hand (common) and plantar warts as having a prevalence of 1.4%; half of all warts that had not been treated resolved within two years [3].

Although over 120 HPV types have been identified, plantar verrucae are usually from HPV types 1, 2, 4, 27 and 57 in healthy children and young adults [3–5]. One study focusing on plantar warts and their relation to demographic and clinical characteristics of 72 patients (including adults) found that the most prevalent genotypes detected among the 105 plantar warts were HPV-57 (37.1%), HPV-27 (23.8%), HPV-1a (20.9%), HPV-2 (15.2%) but a small percentage (2.8%) also had HPV-65 [6]. Most patients (78%) presented with a single plantar wart. It is not uncommon to find multiple HPV types in a single wart [5, 7].

The data for healthy children suggests that, in general, verrucae pedis infection is a mild, self-limiting problem. However, one study reported that nearly 30% of their group considered the warts inconvenient with problems such as pain, irritation and having an unsightly appearance; 38% of the group had attempted treatment of the warts [1]. An earlier study by Ciconte et al [8] identified that plantar warts had similar mean values for psychological morbidity as hand warts. Warts on the hands had higher scores for measures such as “*other people’s view of the lesions*”, but plantar warts had greater scores for measures such as frustration with persistence, pain and impact on activities. Sterling et al [9] identified warts as being cosmetically unappealing, painful and irritating with a further study [10] additionally noting that they cause embarrassment. A recent medical phone-in on the BBC radio featured people reporting that they felt “*humiliated*” when people saw their verrucae and that the verrucae caused “*real misery*” [11]. No study to date has been found that identified the psychological impact of having plantar warts in immunosuppressed patients compared with healthy participants.

The data on the resolution of warts for children who are immunocompromised is less clear. It is apparent that primary immunodeficiency diseases are associated with increased HPV infections and the lesions tend to be more widespread and unusual in presentation [12]. In a study of verrucae pedis in adult participants with a positive and negative HIV (human immunodeficiency virus) status, the same four HPV types were identified regardless of HIV status [13]. An earlier study on verrucae pedis and HIV found that the plantar warts were larger and more numerous when the participant was HIV positive [14]. All participants in these trials were adults. More recent studies on immunocompetent and immunocompromised participants agreed that the same HPV types are found in these populations and otherwise healthy populations [15, 16] but it is still unclear whether the number of HPV types per lesion may differ between immunocompetent and compromised participants [17].

Studies on immunocompromised children are low evidence case reports. Marini et al [18] reported on nine-year-old twin sisters who presented with long-standing severe plantar warts following bone marrow transplantation for Severe Combined Immunodeficiency (SCID). The wart was not HPV typed. Although not referenced, the authors suggest that the prevalence of HPV infection in immunodeficiencies is as high as 40–60% and agree with the picture of typical persistence of the infection rather than the natural history of spontaneous regression, and thus these children will be troubled for longer by lesions that may well be painful and that they also consider unsightly or embarrassing.

Paediatric rheumatic diseases include a group of conditions where autoimmune or autoinflammatory diseases occur. Juvenile Idiopathic Arthritis (JIA) is the most common rheumatic disease of childhood [19]. It is an autoimmune disease whereby antibodies to the body’s synovial structures are generated resulting in joint and soft tissue inflammation. Other conditions include Systemic Lupus Erythematosus (SLE) and Juvenile Dermatomyositis (JDM). In order to suppress or alter the autoimmune reaction, the majority of children are placed on immunosuppressive medication such as methotrexate, and those with more severe disease receive biologic therapies aimed to block specific cytokines in the inflammatory pathway [20]. These children are not immunosuppressed to the degree used in other conditions such as SCID or with organ transplant where the increased incidence of plantar warts and recalcitrant warts has been reported, but anecdotally these children appear to have a greater prevalence of verrucae pedis infections. Prevalence of verrucae pedis has not previously been investigated in this group of children. One study has suggested that, compared to a healthy control group (children with ADHD), children with JIA have a greater risk of opportunistic infections such as *herpes zoster* [21], it is therefore possible that this group is also at risk of developing verrucae and that these may be of a more severe nature with a longer duration. This study aims to identify the prevalence of verrucae pedis in children with JIA and other paediatric rheumatological diseases, to investigate the nature of the verrucae in terms of their duration, resistance to previous treatment, demography and clinical HPV type as well as the patient’s psychological reaction to the presence of verrucae.

## Method

**Fig. 1:**
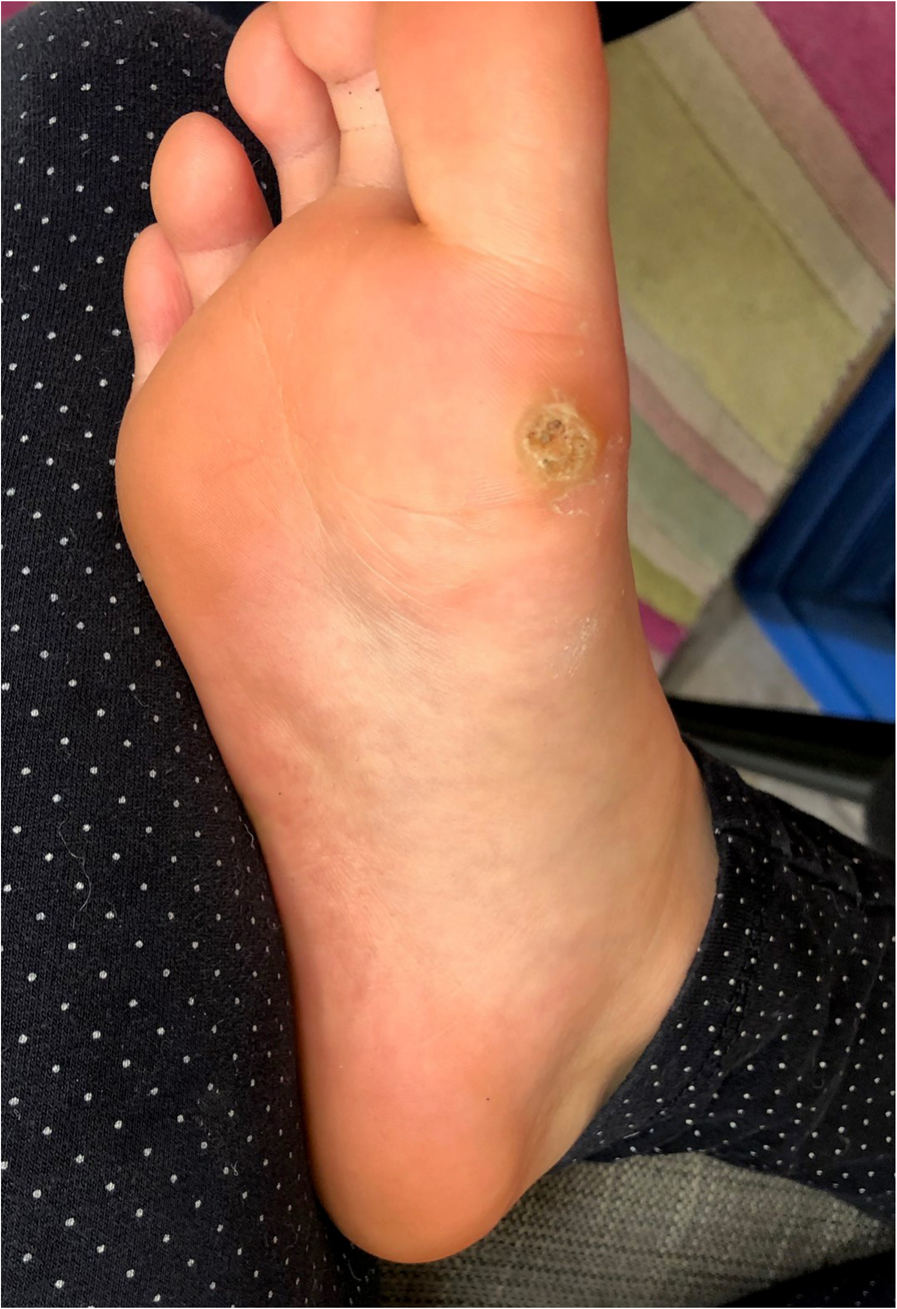
Deep plantar wart (HPV 1)

Agreement for this observational study was provided by the hospital R&D Office who deemed that Ethical Committee consent was not required. Children attending routine out-patient appointments within the paediatric rheumatology department on 10 separate days between Dec 2018 and Dec 2019 were approached to take part in the study**.** The inclusion criteria consisted of children having a rheumatology consultant as the lead clinician and aged between four and 17 years old. The lower age of four years old was set for pragmatic reasons as verrucae pedis are rare in very young children and the time available to undertake the study could be used to focus on the age range when verrucae more typically present. Having a rheumatology consultant as the lead clinician was selected as some children attend the clinics for a rheumatology opinion, for example children with non-inflammatory conditions such as hypermobility or pain syndromes. Non-inflammatory conditions were excluded. Children seeing the rheumatology team and who were within the included age range, were identified from the clinic list when they checked-in for their appointment. Within the reception area, the child and their carer were approached by the researcher was introduced themselves as a member of the rheumatology team. The child and carer were then briefly informed of the nature study. If they were interested in participating, they were then taken to a clinic room and provided with full study information. Informed parental consent and children assent was gained before a visual inspection of the participant’s feet was undertaken to identify potential verrucae; a dermatoscope was used for closer visualisation to confirm diagnosis when necessary. The clinical diagnosis of a verrucae pedis has no gold-standard and the accuracy of the clinical diagnosis is not known. Therefore, diagnosis was confirmed on observation of the typical features such as localised hyperkeratosis, pin-point haemorrhages / capillary thrombi and disruption of dermatoglyphic pattern. Clinical typing of the HPV was determined by appearance [22] but again, the accuracy of this is not known:

Deep plantar wart – single, large verruca with increased overlying hyperkeratosis (typically HPV 1 (see Fig. 1)).

Endophytic wart – small, multiple warts with light hyperkeratosis often occurring in clusters (typically HPV 4).

Mosaic – plaques of verrucae (typically HPV 2).

For those presenting with a verruca, the location/s of the lesions (dorsal, interdigitally and plantar surfaces) were recorded along with the parent recall of onset and duration of presence, previous treatments, parent’s impression of the likely presence of verrucae in other family members and the child was asked about the psychosocial impact “*Does it bother you or worry you that you have a verruca? Why …*? ”. The family’s own understanding of verrucae was sought though asking the same direct question to each participant “*what do you understand a verruca is*?”. Their need for further information on the condition was determined through direct questioning “*Have the doctors here given you any advice?*” and “*Would further advice be useful … … Such as?*”. No specific, validated tool was used to gather the qualitative data.

After the skin assessment, the medical records were reviewed to confirm the participant met the inclusion criteria as some patients did not know their diagnosis and some patients were attending an appointment for a second opinion whereby the rheumatologist was not the lead consultant. Participant information obtained from the medical records included age, diagnosis and medication taken. No personal identifying information was recorded.

The same researcher assessed all participants and was known to some of the participants having worked within the rheumatology team as a podiatrist for 25 years and has specialised in paediatric foot conditions for this time. The data was extracted and managed by the researcher.

### Data analysis

Data is represented using descriptive statistics to allow visualisation of the raw data and to make simple interpretations. Inferential statistics have been applied to make generalisations about the larger population from which this sample is drawn and determine whether observed differences (presence / absence of verrucae) have occurred by chance. The odds ratio has been applied as it is used to compare the relative odds of the occurrence of the outcome of interest (ie. verruca pedis) given the exposure to the variable of interest (ie. medication). Non-significant outcomes may result from the small sample size of the study.

For analysis, participants were separated into age groups of children (4–12 years) and teenager (13–17 years) with the children’s group being divided to allow for comparison better younger and older children (4–8 / 9–12 years).

## Results

A total of 77 children were invited to take part in the study. Two children meeting the inclusion criteria declined to take part in the study (too embarrassed (1); not interested (1)) and four were excluded after review of their medical records showed that their conditions did not meet the inclusion criteria (autoimmune thyroid disease, widespread musculoskeletal pain, undiagnosed condition, (non-inflammatory) pain syndrome).

The study included 71 children; ages ranged from four to 17 years old (mean = 11 years; SD = 3.5 years). Seventy three percent of children had a diagnosis of Juvenile Idiopathic Arthritis. The conditions included and disease-modifying medications taken are shown in Table 1.

**Table 1:** Showing diagnosis and medication prescribed in the study population

Disease type (***n***=)	Medication
Juvenile Idiopathic Arthritis	
• Systemic (5)	MTX (2); Biologic only (2); MTX + Biologic (1)
• Oligo (13)	MTX (7); MTX + Biologic (2); no meds (4)
• Extended Oligo (11)	MTX (6); MTX + Biologic (4); no meds (1)
• Poly (17)	MTX (10); MTX + Biologic (5); Biologic only (1); no meds (1)
• Enthesitis Related Arthritis (5)	MTX (1); MTX + Biologic (1); no meds (2); sulfasalazine (1)
• Psoriatic (3)	MTX (2); Sulfasalazine (1)
• Mono (1)	No meds (1)
• Unclassified (2)	MTX (1); MTX + Biologics (1)
Juvenile Dermatomyositis	
JDM overlap (1)	MTX + Prednisolone (1)
Vasculitides (2)	Azothiaprine (1); no meds (1)
Fever Syndromes (5)	Colchicine (2); Prednisolone (1); No meds (2)
Immunopathies (2)	Immunoglobulins (1); No meds (1)
Connective tissue disease (1)	No meds (1)
Uveitis (2)	MTX (1); Mycophenolate mofetil (1)
Other (1)	MTX + Biologic (1)

Abbreviations: *MTX* Methotrexate

Of the group, 55 children had no verrucae present, 16 children had one or more verrucae. The prevalence of verrucae in the whole group was 22.5%. Table 2 shows the prevalence of verrucae by disease and by disease subgroup for the JIA group.

**Table 2:** Showing the prevalence of verrucae pedis within each disease type

Disease type	Percentage of specific group with verrucae pedis
**Juvenile Idiopathic Arthritis**	**22.9%**
• Systemic	• 0%
• Oligo	• 1.8%
• Extended Oligo	• 12.3%
• Poly	• 8.8%
• Enthesitis Related Arthritis	• 0%
• Psoriatic	• 0%
• Mono	• 0%
• unclassified	• 0%
**Total Non-Juvenile Idiopathic Arthritis Participants**	**21.4%**

Methotrexate was the most frequently prescribed medication. The chance of having a verruca was greater when taking methotrexate plus a biologic rather than methotrexate alone (OR = 4.3, 95%CI 1.26 to 14.9, *p* = 0.026). But overall, there appeared to be no greater chance of having verrucae if taking immunosuppressive medication than compared to having no medication (OR = 1.1, 95%CI 0.26 to 4.48, *p* = 0.46). Table 3 shows the presence / absence of verrucae by medication taken.

**Table 3:** Showing number of Verrucae Pedis present in relation to medication prescribed

Medication prescribed	Verrucae Pedis present	Verrucae Pedis absent
None	3	11
Methotrexate	4	26
Methotrexate + biologic	6	9
Methotrexate + prednisolone	0	1
Biological only	1	2
Prednisolone	1	0
Sulfasalazine	0	2
Azathioprine	1	0
Mycophenolate mofetil	0	1
Immunoglobulins	0	1
Colchicine	0	2

Only one participant associated the onset of their verrucae with the start of taking medication (Tocalizamab being added to Methotrexate) and one participant associated their verrucae with the start of a swimming programme. None of the other participants identified a triggering factor.

Children with verrucae tended to be 9 years old or older and most children had one to three verrucae. Fewer children in the older age range (13–17 years) had verrucae and of those teenagers with verrucae, very few had recent lesions. In total, 37.5% of the young people had their verrucae for 24 months or more. Table 4 demonstrates these figures.

**Table 4:** Subject age range and duration of VPs when present

Age range	Number without VP	Number with VPs	VP duration		No. of VPs present per subject	
					1–3	+ 4
4–8 years	16	2	< 6 m	1	2	
			6 m			
			6–12 m			
			12 m			
			18 m			
			24 m			
			> 24 m	1		
9–12 years	16	9	< 6 m		7	2
			6 m	2		
			6–12 m	1		
			12 m	3		
			18 m	1		
			24 m	1		
			> 24 m	1		
13–17 years	23	5	< 6 m		4	1
			6 m	2		
			6–12 m			
			12 m			
			18 m			
			24 m			
			> 24 m	3		

All verrucae occurred on the plantar surface with none seen on the dorsum of the digits or in the interdigital spaces. The deep plantar wart (HPV 1) was the most frequently seen verruca type, being present in 12 of the 26 individually counted verrucae. When this type was identified only one or two lesions were present per foot in 75% of the cases. Endophytic verrucae (HPV 4) were counted in seven verrucae and one case where there were multiple endophytic verrucae present; five identifiable mosaic verrucae (HPV 2) were seen with one further case having multiple plaques. Only four children with verrucae reported that they caused some pain, the remaining 12 children had no pain from the lesions.

When asking whether any other family member in the household had verrucae, there were three cases (18.75%) of the children with verrucae reporting someone else in the household having with verrucae, compared to three children without verrucae (7%).

When the child had a verruca, the parent and child were asked what their understanding of a verruca. Less than half the participants with a verruca knew that it was a wart (*n* = 7), of the remaining nine participants, three were not aware of what a verruca was, three recognized it was a type of infection (virus / germ / contagious spot) and three participants thought it was hard skin or dirt. Despite not being either not entirely clear on what the lesions were or not experiencing any discomfort from the verruca, the majority of participants (81%) had sought treatment for the verrucae. This consisted of over the counter (OTC) remedies in ten cases (typically Bazuka gel which contains 12 to 26% salicylic acid), one was advised by pharmacist to watch & wait, one by a GP to “*rub it with a stone*”, and one had tried laser treatment. None of the participants mentioned seeking podiatric care. Of the 13 participants trying treatment, only the laser treatment was considered, by the participant, to be successful although that participant still had verrucae present on observation. All but two participants expressed a wish for further information about verrucae and their treatment to be available in the clinic.

When considering the psychosocial impact of having a verruca, two-thirds of the participants were not concerned about the lesion being present, four said they “*did not like them*” being present, one participant said they were “*embarrassed*” by them and one participant said it “*made their feet look dirty and not looked after properly*”.

## Discussion

The study has identified that the prevalence of verrucae in children with JIA and other paediatric rheumatological conditions is 22.5% which is very similar to the prevalence identified in the most recent large study (21%) of school children aged four to 12 years of age [1]. Unlike other conditions where the immune system is suppressed or compromised and a much higher prevalence of warts have been identified, in children with rheumatic conditions there appears to be no greater risk of developing verruca pedis infection than is seen in the healthy population. The prevalence of verrucae pedis also reduce from young children into the teenage years suggesting that in most cases the immune system can recognise and resolve these infections. However, in this group and particularly in the teenagers, 37.5% of children had verrucae that had been present for longer than two years. This is only slightly more than would be expected to be seen in an otherwise healthy population where approximately 33% of cases would be expected to continue beyond two years [2]. Massing and Epstein [2] is the only study that measured prevalence beyond one year however their study included an institutionalised population such that environmental situations were very different between this and the current study.

The peak prevalence of verrucae was found in the nine- to 12-year-old age group and the majority had only 1–3 verrucae which also shows similarity to the typical childhood population [1, 5]. The most frequently presenting verrucae was the deep plantar wart which occurred as a single lesion. This has previously been linked to HPV 1 infection [22] however Bruggink et al. [5] identified the most prevalent HPV types in single warts (including hand warts) as being from types HPV 27 (24%), HPV 57 (22%), HPV 2 (22%) and HPV 1 (19%). Despite the variety of HPV types presenting as a single lesion, their study found that HPV 1 showed a distinct profile with it being found in children of less than 12 years old, presenting with four or fewer warts and occurring on the plantar surface and being of less than six months duration. The single verruca seen in the paediatric rheumatology participants fits this profile of the most common lesion seen in the healthy childhood population.

In the current study there was no increased risk of having a verruca when a family member was affected by verrucae. In the past, verruca infection was associated with swimming pools [23] and then more specifically to shower and locker room areas [24]. More recently Van Haalen et al. [25] found that environmental factors connected to barefoot activities such as public showers or swimming pool were not related to the presence of warts but were related to family members or other members of the school class having warts. The study found an increased risk of the presence of warts when a family member had warts (OR 1.9, 95%CI 1.3–2.6) and agreement was found in a further study by Bruggink et al [26] which calculated a Hazard Ratio of 2.08 (95% CI 1.52–2.86) when a family member had verruca infection. Both studies were much larger study than the current study and so differences between studies may be related to the small sample size of the current study. It might be argued that children with rheumatic conditions may be participating in sports and barefoot activities less than healthy children thus having less peer-barefoot contact, however it is unlikely that their condition is impacting on the amount of barefoot-contact time they are having in their own households.

Gender was not specifically considered as a risk factor within this study. Although there is some suggestion in the literature that verrucae pedis may be more commonly found in females, the earlier prevalence studies by Van Haalen et al [25] and Bruggink et al [1] do not support this. Paediatric rheumatic conditions including JIA have a strong predilection for girls [27] and so comparisons between the sexes in this small study is not undertaken.

Although being on medication in general did not increase the chance of having verrucae in this group of children, there was an increase chance of having verrucae when on biologic agents. This was seen in children taking methotrexate and a biologic therapy compared to methotrexate alone. This may be due to the small numbers involved in the study and care does need to be taken when accepting this result due to the small sample size. Methotrexate inhibits DNA synthesis thus reducing the body’s ability to generate an auto-immune response. It will therefore not be able to generate a full immune response to invading organisms and as such live vaccines should not be given to patients on methotrexate, and susceptibility to viral warts in a listed side effect (https://www.ouh.nhs.uk) [28]. However, by the very fact that DNA synthesis is reduced by methotrexate, the verruca may move into a latent state and although not resolving or multiplying, will remain in the epidermis for longer periods than seen in a healthy population. The biologic therapies often target TNF-α which plays a critical role in the control of viral infection since it is involved in recruiting and activating macrophages, Natural Killer, T cells, and antigen presenting cells [29]. Reduction of TNF-α by treatment with TNF-α blockade may increase the risk of developing a viral infection such as HPV [29] or not being able to resolve an infection when it occurs. One case study has reported on extensive verrucae pedis infection in a 17-year-old girl receiving etanercept for treatment of JIA [30]. The verrucae resolved soon after the medication was stopped.

The children in the current study showed the same psychological concerns about having a verrucae pedis infection and expressed their feelings using similar terms, such as feeling the feet were dirty or being embarrassed by the lesions. The numbers of young people reporting these concerns were similar between this and another study (33% vs 30% respectively) [1]. However, in the current study it was more typical for the parents to seek treatment of the verrucae (81%) compared to the healthy population, where only 38% had sought treatment in the Bruggink study [1], and 87.5% of those with verrucae present wanted further information on the condition. This might reflect the heightened medical concerns of the parents of children with long-term conditions.

### Limitations

This was a small study and therefore care should be taken in generalizing the results to the larger population. In order to improve the method of epidemiological data collection, the aim was to gather data from all children at these clinics, who met the inclusion criteria, this was not possible as patients often left the clinical area before the researcher was available to recruit them. The diagnosis of the verruca was not difficult in this clinical situation but the accuracy of the clinical diagnosis without biopsy confirmation should be considered, particularly concerning the typing of the verrucae. Although all participants were asked the same questions to determine their understanding of the nature of verrucae, and regarding the emotional impact of the lesion, the questions asked were had not been validated nor was a recognized score used and therefore the ability of these questions to reflect the true situation needs to be considered. Future studies could include using a quality-of-life questionnaire such as the Children Dermatology Life Quality Index [31], a validated tool. This would seem appropriate given the answers to the single question asked in this study would align to the question within that questionnaire “Over the last week, how embarrassed or self-conscious, upset or sad have you been because of your skin?”. However other questions on that score may not apply and limit the range of outcomes scores achievable and no tool has been validated for verrucae specifically.

Some of the data gathered required the parents / child to recall past events or timing. Such questions often created disagreement between the parties but typically, with discussion, events were clarified but may still have been subject to recall bias. Future studies could be improved by the inclusion of a control group to allow direct comparison to an aged-matched population without a rheumatological diagnosis.

## Conclusion

This is the first study to evaluate the prevalence of verrucae pedis in a paediatric rheumatological population. Children with JIA and other rheumatic conditions have no greater prevalence of verrucae pedis compared to the general paediatric population. The verrucae present were of a similar clinical type and did not seem to be more widespread or have a longer-standing duration than has been reported in other immunocompromised populations. Verrucae pedis prevalence peaked in the 9–12 years age group with teenage participants showing a low prevalence. There was a slight increased percentage of lesions remaining beyond 24 months than has been reported in other healthy populations. The children in this study seemed to be less concerned psychologically about their verrucae, despite this most families had sought treatment for the verrucae and reported that they would value further information about the condition. Podiatrists can therefore apply their general knowledge regarding verrucae to this specific group children when providing information to patients, particularly in terms of spontaneous regression and risk of developing verrucae. The management of verrucae in children with rheumatic conditions was not considered in this study so any differences in response to treatment in this group is not known.

## Data Availability

Data and materials used and/or analysed during the current study are available from the corresponding author on reasonable request.

## Abbreviations

*VP*: Verruca Pedis
*JIA*: Juvenile Idiopathic Arthritis
*HPV*: Human Papilloma Virus
*HIV*: Human Immunodeficiency Virus
*SLE*: Systemic Lupus Erythematosus
*JDM*: Juvenile Dermatomyositis
*SCID*: Severe Combined Immunodeficiency
*ADHD*: Attention Deficit Hyperactivity Disorder
